# Correlating estrogen replacement therapy and temporomandibular disorders: a comprehensive review following PRISMA principles and cochrane handbook for systematic reviews of interventions

**DOI:** 10.1186/s12903-023-03697-2

**Published:** 2024-01-16

**Authors:** Mohammad Khursheed Alam, Maysara Adnan Ibrahim, Manal Jamil Almaslamani, Musab Hamed Saeed, Yuliia Siurkel, Vincenzo Ronsivalle, Marco Cicciù, Giuseppe Minervini

**Affiliations:** 1 Preventive Dentistry Department, College of Dentistry, Jouf University, 72345 Sakaka, Saudi Arabia; 2 Department of Dental Research Cell, Saveetha Institute of Medical and Technical Sciences, Saveetha Dental College and Hospitals, Chennai, 600077 India; 3 Department of Public Health, Faculty of Allied Health Sciences, Daffodil International University, Dhaka, 1207 Bangladesh; 4 Department of Preventive and Restorative Dentistry, College of Dental Medicine, University of Sharjah, Sharjah, Saudi Arabia; 5 Clinical Sciences Department, College of Dentistry, Ajman University, Ajman, UAE; 6 Center of Medical and Bioallied Health Sciences Research, Ajman University, Ajman, UAE; 7 International European University School of Medicine, Akademika Hlushkova Ave, 42B, Kiev, 03187 Ukraine; 8 Department of Biomedical and Surgical and Biomedical Sciences, Catania University, 95123 Catania, Italy; 9 Saveetha Dental College and Hospitals, Saveetha Institute of Medical and Technical Sciences (SIMATS), Saveetha University, Chennai, Tamil Nadu India; 10 Multidisciplinary Department of Medical-Surgical and Odontostomatological Specialties, University of Campania “Luigi Vanvitelli”, 80121 Naples, Italy

**Keywords:** Estrogen replacement therapy, Temporomandibular disorders, Menopause, Hormone replacement therapy, Craniomandibular, Clenching, Bruxism, Myofascial pain, Cortisol, Sleep disorders, TMD

## Abstract

**Background:**

Estrogen replacement therapy (ERT) is a common hormonal treatment for postmenopausal women, aimed at alleviating menopausal symptoms and reducing the health risks associated with estrogen deficiency. However, the impact of ERT on temporomandibular disorders (TMDs) remains unclear. This systematic review aims to evaluate the relationship between ERT and TMDs, including TMD occurence, pain, and associated symptoms.

**Methods:**

A comprehensive search of seven electronic databases was conducted using predefined search terms and Boolean operators. Inclusion criteria encompassed studies examining the association between ERT and TMDs. Two independent reviewers screened the identified articles, extracted data, and assessed the risk of bias using the RoB -2 tool.

**Results:**

Search strategy identified a total of 3 articles which met the inclusion criteria. The included studies investigated the impact of ERT on TMD occurrence and its related symptoms. The analysis revealed no significant association between ERT and TMD occurrence. A significant dose relationship was noted in one of the studies while another mentioned the possible relationship of TMD with educational status. Risk of bias among the studies was low, and the overall quality of evidence was deemed to be high.

**Conclusion:**

This systematic review suggests that there is no conclusive evidence supporting an increased risk of TMDs among women receiving ERT. The findings indicate that ERT is unlikely to have a noticeable impact on TMDs. However, due to the limited number of studies available, further research is warranted to strengthen these conclusions and explore potential factors that may influence the relationship between ERT and TMDs.

## Introduction

Temporomandibular disorders (TMDs) are a group of conditions affecting the temporomandibular joint and associated structures, characterised by pain, dysfunction, and impaired quality of life [1–8]. Common symptoms include jaw pain or tenderness, often exacerbated by chewing or jaw movement. Individuals with TMD may experience limitations in jaw movement, such as difficulty opening the mouth widely or a sensation of the jaw getting stuck. Clicking, popping, or grating sounds during jaw movement may also occur. Sometimes, TMD can also manifest as facial pain, headaches, and discomfort in the neck and shoulders, reflecting the intricate interplay between the temporomandibular joint and surrounding musculature. These symptoms can significantly impact daily activities such as eating, speaking, and facial expressions, emphasising the importance of timely diagnosis and management. These disorders pose a significant health burden, with one study indicating that nearly 80% of the female participants in their study required treatment due to TMDs. TMDs primarily affect women, particularly those in their reproductive and postmenopausal years, suggesting a potential role for hormonal factors in the development and progression of these disorders [9] (Table 1).

**Table 1:** Abbreviations used in this review

Term	Abbreviation used
Estrogen replacement therapy	**ERT**
Hormone replacement therapy	**HRT**
Craniomandibular index	**CMI**
Odds ratio	**OR**
Risk ratio	**RR**
Control group	**CG**
Confidence interval	**CI**
Temporomandibular disorders	**TMD**
Research Diagnostic Criteria for Temporomandibular Disorders	**RDC/TMD**
Postmenopausal	**PM**
Visual analogue scale	**VAS**
Temporomandibular joint	**TMJ**

Estrogen replacement therapy (ERT) is a commonly prescribed hormonal therapy for postmenopausal women to alleviate menopausal symptoms and mitigate the health risks associated with estrogen deficiency [10]. ERT can be administered in various forms, including oral tablets, transdermal patches, and vaginal creams. In addition to estrogen, some women may also receive progestin as part of their hormone replacement regimen. Progestin is a synthetic form of progesterone, a naturally occurring hormone in the female body [11]. Both estrogen and progestin play distinct roles in HRT and contribute to the overall hormonal balance in postmenopausal women [12].

Estrogen, the primary female sex hormone, exerts a wide range of effects throughout the body. It plays a crucial role in maintaining reproductive health, bone density, and cardiovascular function [12]. Estrogen receptors are present in various tissues, including the ovaries, uterus, breasts, brain, and bones. By binding to these receptors, estrogen regulates gene expression and modulates cellular activity [13]. In the context of HRT, estrogen helps alleviate menopausal symptoms such as hot flashes, vaginal dryness, and mood swings. It also helps prevent osteoporosis by promoting bone formation and inhibiting bone resorption [13]. Furthermore, estrogen has beneficial effects on lipid metabolism, blood vessel dilation, and cognitive function [14].

Progestin, on the other hand, is primarily used in combination with estrogen in HRT regimens to provide additional benefits and mitigate certain risks associated with estrogen alone [15–19]. Progestin acts on the endometrium (the lining of the uterus) and helps prevent endometrial hyperplasia and the development of uterine cancer in women with an intact uterus. This is particularly important because unopposed estrogen therapy can increase the risk of endometrial hyperplasia and cancer [20]. Progestin provides a protective effect by counteracting the proliferative effects of estrogen on the endometrium. By adding progestin to the HRT regimen, the risk of endometrial cancer can be effectively minimized [20].

Beyond its effects on menopausal symptoms and bone health, estrogen exerts influences on numerous physiological processes throughout the body [21]. Estrogen receptors are present in various tissues, including the reproductive organs, brain, cardiovascular system, and musculoskeletal system [13]. This widespread distribution of estrogen receptors suggests that estrogen has multifaceted actions and may impact diverse aspects of health and well-being [13].

However, the impact of ERT on TMDs remains controversial. Estrogen replacement therapy can impact TMDs in several ways when analysed on molecular levels. The first mechanism could be the hormonal influence on connective tissues. Estrogen is known to have an impact on the synthesis and metabolism of collagen and other extracellular matrix components. TMD often involves changes in the temporomandibular joint (TMJ) and surrounding tissues. Fluctuations in estrogen levels could influence the structural integrity and remodelling of these tissues. The second possible mechanism is the role of estrogen in inflammatory responses. Estrogen has immunomodulatory effects and can influence inflammatory responses. Inflammation is implicated in TMD, and hormonal fluctuations may contribute to the inflammatory processes in the temporomandibular joint and associated structures. ERT can also affect neurotransmitter regulation and thereby affect TMDs. Estrogen receptors are present in the central nervous system, including areas related to pain modulation. Changes in estrogen levels may affect neurotransmitter systems involved in pain perception, potentially influencing the experience of pain associated with TMD. Some studies have suggested that ERT may increase the risk of TMDs, possibly through its influence on hormonal balance and connective tissue metabolism [22–25]. On the other hand, conflicting evidence exists, with studies reporting no significant association between ERT and TMDs. Despite the potential clinical implications, there is currently no comprehensive review summarizing the available evidence on this topic in terms of qualitative statistical synthesis. Therefore, this systematic review aims to address this gap in knowledge by systematically examining the literature and synthesizing the evidence regarding the association between ERT and TMDs. By conducting a comprehensive search across multiple databases and employing rigorous inclusion and exclusion criteria, this review aimed to identify and analyze relevant studies that have investigated the impact of ERT on TMD occurrence and the presence of pain and symptoms related to TMDs.

## Materials and methods

### Eligibility criteria

The PICOS (population, intervention, comparison, outcome, study design) strategy employed for the study was.

#### Population

The population of interest was female (women), irrespective of their age.

#### Intervention

The intervention of interest was ERT. This encompassed the administration of exogenous estrogen, including various forms such as oral medications, transdermal patches, or injections.

#### Comparison

The comparator group was individuals who did not receive ERT. This could include individuals receiving a placebo, individuals not undergoing HRT, or those undergoing alternative treatments for the same medical conditions.

#### Outcome

The primary outcome of interest was the risk or incidence of TMDs. This included various aspects of TMD, such as signs and symptoms, pain, dysfunction of the TMJ, or related psychosocial factors.

#### Study design

The study designs considered were randomised controlled trials (RCTs) and case–control studies.

The inclusion and exclusion criteria for this study were carefully established to ensure the selection of relevant and high-quality studies.

#### Inclusion criteria

1. Study Design: Only original research articles published in peer-reviewed journals
2. Population: The study population consisted of individuals who were receiving ERT or HRT for various indications or conditions.
3. Intervention: The primary intervention of interest was ERT or HRT, either in the form of exogenous estrogen or a combination of estrogen and progestin.
4. Outcome: The main outcome of interest was the occurrence, prevalence, or incidence of TMDs. Studies reporting relevant measures of TMDs, such as clinical diagnosis, symptomatology, or validated assessment tools, were included.
5. Language and Publication Date: No language or publication date restrictions were imposed to ensure a comprehensive search and inclusion of relevant studies.

#### Exclusion criteria

1. Animal Studies: Studies conducted on animal models or in vitro experiments were excluded.
2. Review Articles and Case Reports: Review articles, systematic reviews, meta-analyses, and case reports were excluded from the review. However, the reference lists of relevant review articles were screened for potential inclusion.
3. Irrelevant Studies: Studies that did not specifically investigate the association between ERT and TMDs were excluded.
4. Duplicate Studies: Duplicate studies or redundant publications from the same study were excluded to avoid duplication of data.

### Search strategy

This review follows the PRISMA (Preferred Reporting Items for Systematic Reviews and Meta-Analyses) protocol [26] and is registered with PROSPERO (CRD42023439749).

Seven databases were comprehensively searched for relevant articles. Boolean operators (AND, OR) and MeSH (Medical Subject Headings) keywords were employed to construct the search strategy and capture the desired literature. The search strategy utilised a combination of keywords and MeSH terms related to ERT and TMDs, ensuring the inclusion of relevant articles. The search strategy employed boolean operators (AND, OR) to combine the keywords and MeSH terms effectively, as seen in Table 2.

**Table 2:** Search strategy implementation across different databases

Database	Search Terms
PubMed/MEDLINE	(estrogen[MeSH Terms] OR hormone replacement therapy OR HRT) AND (temporomandibular joint disorders[MeSH Terms] OR TMD OR TMJ disorders)
Embase	(estrogen OR hormone replacement therapy OR HRT) AND (temporomandibular joint disorder OR TMD OR TMJ disorder)
Cochrane Library	(estrogen OR hormone replacement therapy OR HRT) AND (temporomandibular disorder OR TMD OR TMJ disorder)
Scopus	(estrogen OR hormone replacement therapy OR HRT) AND (temporomandibular disorder OR TMD OR TMJ disorder)
Web of Science	(estrogen OR hormone replacement therapy OR HRT) AND (temporomandibular disorder OR TMD OR TMJ disorder)
PsycINFO	(estrogen OR hormone replacement therapy OR HRT) AND (temporomandibular disorder OR TMD OR TMJ disorder)
CINAHL	(estrogen OR hormone replacement therapy OR HRT) AND (temporomandibular joint disorder OR TMD OR TMJ disorder)

### Data extraction

Two reviewers ensured the meticulous and reliable extraction of data from selected studies. A standardised data extraction form was developed, encompassing various key aspects such as study characteristics, participant demographics, intervention details, outcome measures, and other relevant data points. The data extraction process was conducted independently by each reviewer. In cases of uncertainty or disagreement, consensus meetings and discussions were held among the reviewers to reconcile discrepancies and reach a consensus on the extracted data.

### Quality assessment

The ROB-2 (Risk of Bias in Randomised Studies of Interventions) tool was employed to assess the quality of the studies included [27]. Two reviewers independently evaluated each included study to identify potential sources of bias across several domains. The reviewers evaluated each domain according to pre-defined criteria and guidelines provided by the tool, assigning a judgement of low, high, or critical risk of bias. In cases of disagreement or uncertainty, consensus meetings were held to reach a collective decision.

## Results

### Study characteristics

**Fig. 1:**
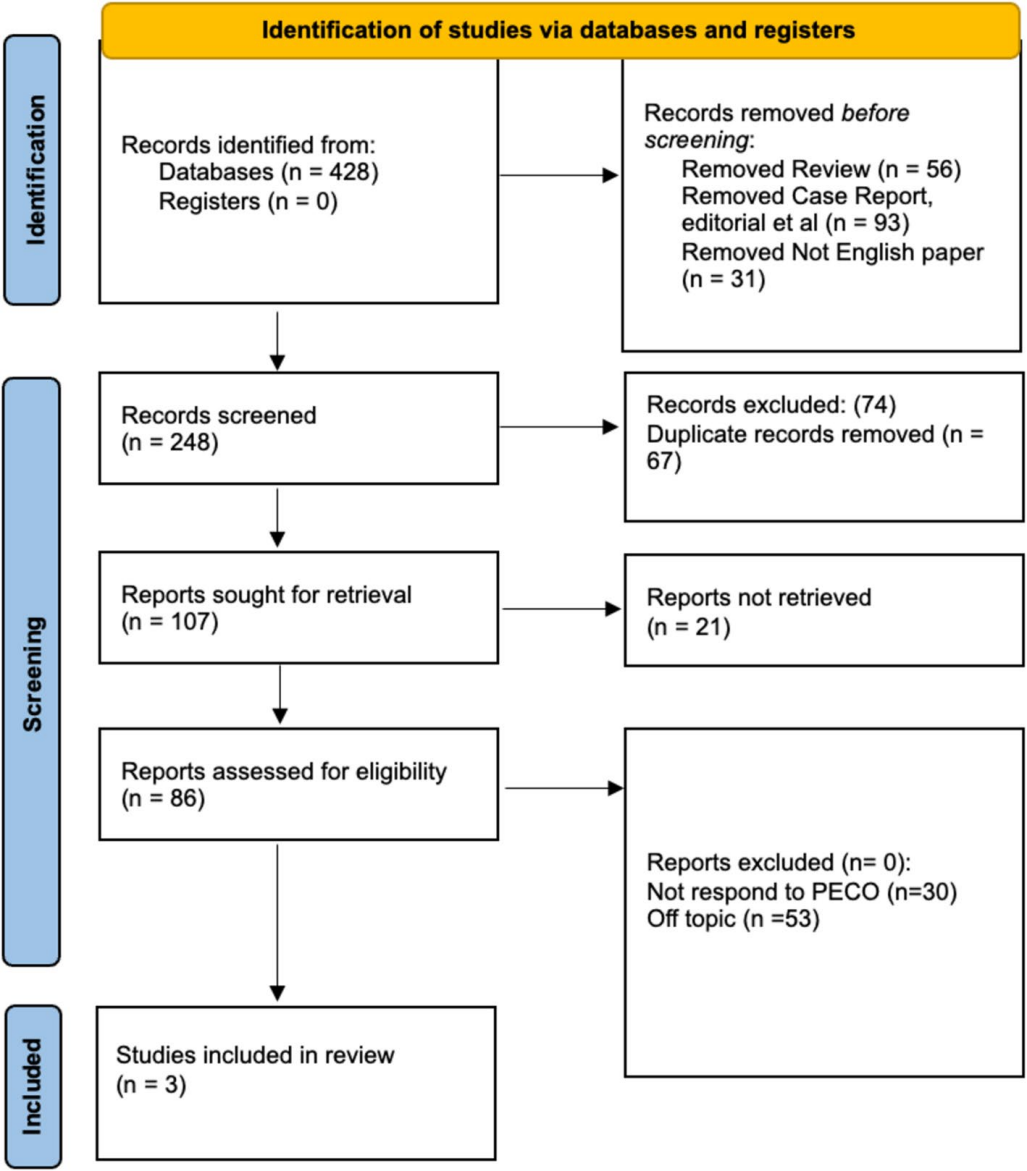
Graphical representation of the PRISMA guideline utilisation in the review

Of the 428 studies screened, only three studies fulfilled the eligibility criteria and were involved in the final analysis (Fig. 1).

Table 3 provides an insight into the study IDs, publication year, location of the study, sample size, and age ranges of the participants.The studies included are Hatch et al. [26], LaResche et al. [27] and Nekora et al. [28]. Two studies were conducted in the USA and one in Turkey. The study sample ranged from 91 to 1291. The majority of the study population was in the 4^th^ or 5^th^ decade. The study by Nekora et al. was conducted on post-menopausal women.

**Table 3:** Assessment of demographic variables selected for the review

Study ID	Year	Region	Sample size (n)	Age range (in years)
**Hatch et al. **[26]	2001	USA	174	37–82
**LeResche et al. **[27]	1997	USA	1291 (post -menopausal)	. > 40
**Nekora et al. **[28]	2008	Turkey	91 (post-menopausal)	52.51 ± 5.26

### Main findings

Table 4 provides an overview of the studies included in the analysis, such as study design, groups assessed, TMD assessment techniques, HRT types used, inferences, and additional inferences. Overall, two studies showed no difference in TMD occurrence with oestrogen replacement, while one showed a definite association between the variables. This suggests that there is no conclusive evidence of HRT for the occurrence of TMD or its possible risk.

**Table 4:** Assessment of technical variables selected for the review

Study ID	Protocol	Groups assessed	TMD assessment technique	HRT type used	ERT assessment inferred	Additional inferences
**Hatch et al. **[26]	Case control	HRT (*n* = 174) and CG (*n* = 336)	CMI, DI and MI	Estrogen in the form of esterified estrogens, estradiol, estropipate, estrone and conjugated estrogens. (exogenous)	Estrogen use did not place women at increased risk of developing TMDs	It was observed that women on estrogen replacement were better educated, had higher income and lived in suburban areas as compared to the control group
**LeResche et al. **[27]	Case control	HRT (*n* = 1291) and CG (*n* = 5164)	Not applicable as cases here were patients referred for TMD pain	Estrogen	An odd of 1.77 (95% CI; 1.53–2.06) was noted for TMD referrals in estrogen users versus non users, significant at *p* = 0.0001	A significant dose response relationship was noted. An increased + odds for TMD referral was noted in women consuming 185 mg of yearly cumulative estrogen dosage
**Nekora et al. **[28]	Case control	HRT (*n* = 91) and CG (*n* = 89)	Clinical examination based on Dworkin and LeResche	Estradiol and conjugated estrogen if estrogen was prescribed; Medroxyprogesterone acetate and norethisteron Acetate if progestin was prescribed	No significant difference was noted for pain in TMJ or surrounding musculature and joint sounds between the HRT group and CG was observed	Women on HRT had a greater proportion of high school education

The study by Hatch et al. [26] indicated that the use of exogenous estrogen in HRT did not increase the risk of developing TMD. This finding implies that women who underwent estrogen replacement therapy are not more likely to experience TMD compared to those who do not receive HRT. The analysis also considered other factors such as socioeconomic status, lifestyle, and healthcare utilization. Similarly, the study by Nekora et al. [28] found no significant difference in the signs and symptoms of TMD between the HRT group and the control group. This suggests that HRT, regardless of the specific type used, does not seem to influence the occurrence of TMD symptoms in the population studied. In contrast, the study by LeResche et al. [27] reported a higher odds ratio among estrogen users compared to non-exposed individuals, suggesting a potential association between estrogen-based HRT and TMD. Additionally, a dose–response relationship was observed, indicating that higher estrogen exposure may further increase the risk of TMD. The study also found that oral contraceptive use was associated with an increased risk of TMD, approximately 20% higher. These findings suggest a potential role of specific HRT formulations, such as progestin and estrogen combinations, in influencing TMD risk.

### Risk of bias

**Fig. 2:**
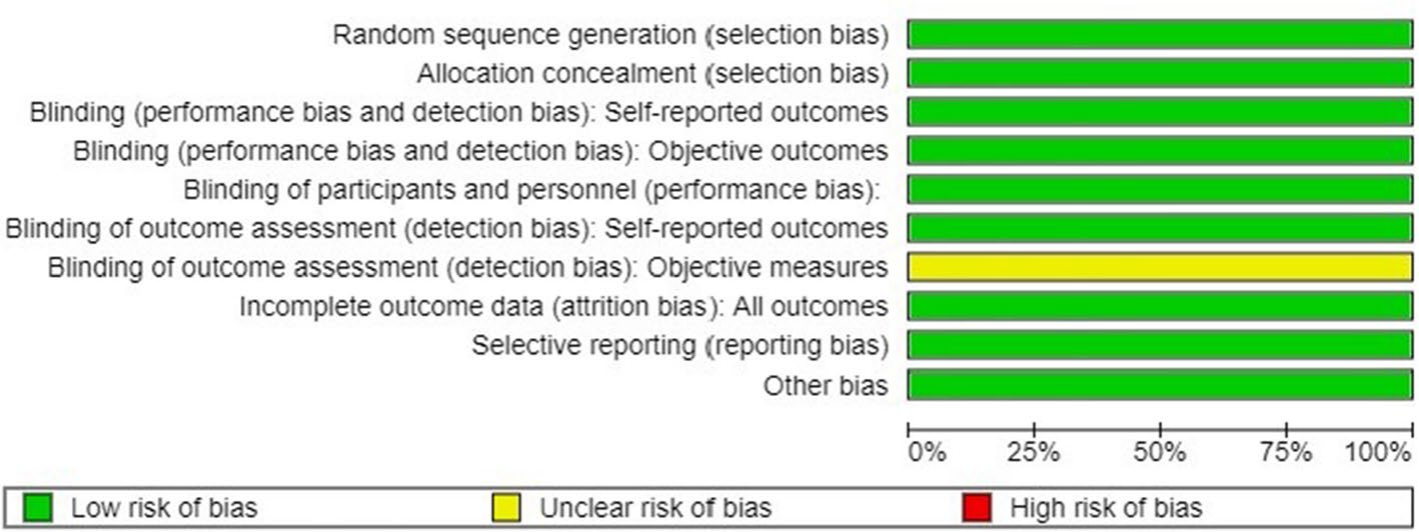
Risk of bias graph of included studies

**Fig. 3:**
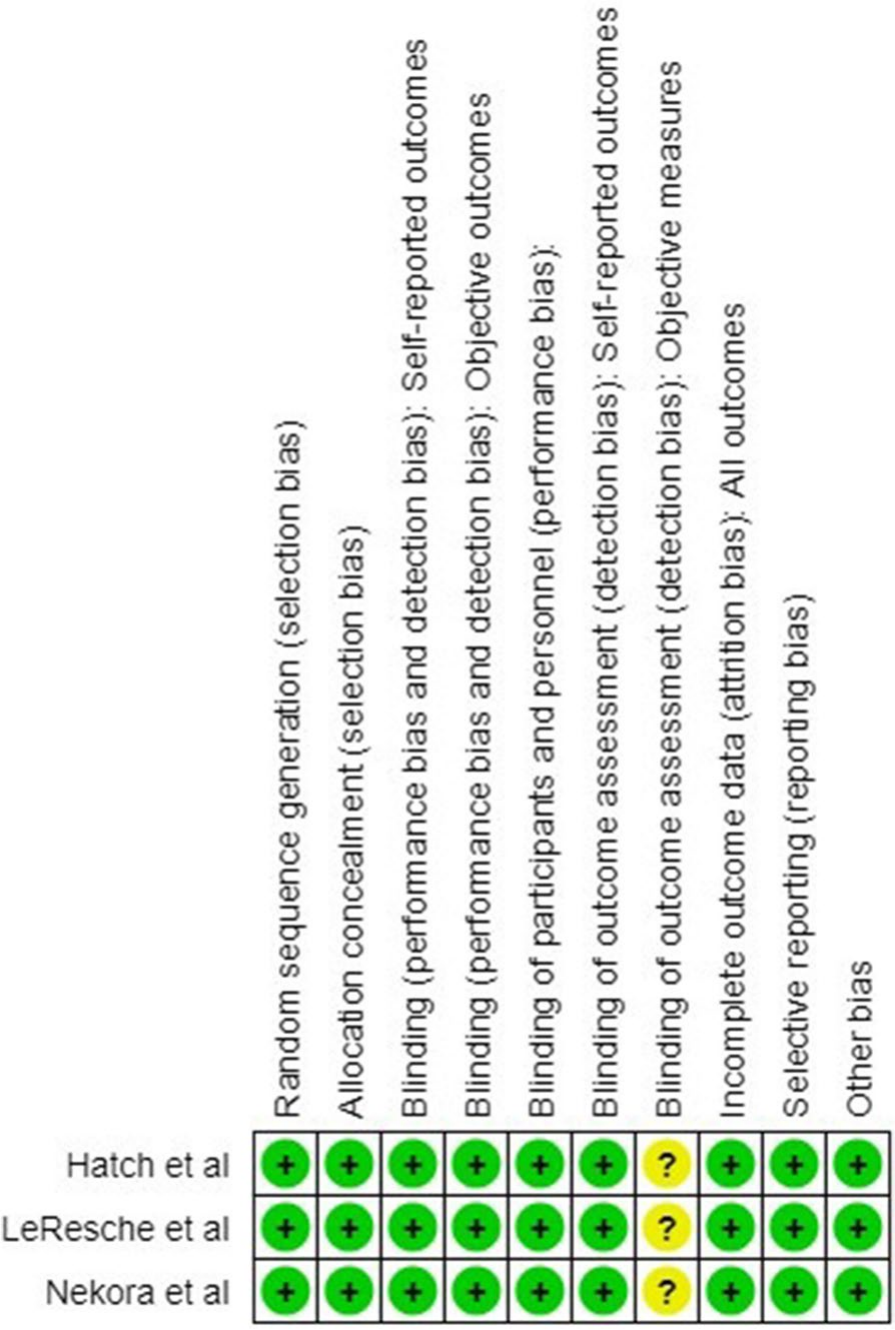
Risk of bias summary of included studies

The studies were represented as being of high quality as assessed by the ROBINS-1 tool, as seen in Figs. 2 and 3. The only domain that raised concern in all three studies was outcome assessment objectively, as none used the standard criteria for TMD evaluation, such as DC/TMD.

The study of LeResche et al. [27] included participants who were referred for TMD related symptoms. The study of LeResche et al. [27] and Nekora et al. [28] included only postmenopausal women on ERT. Hatch et al. did not specify about menopause criteria, but the authors conducted a sensitivity analysis based on age groups to differentiate between ovulating and non-ovulating women.

## Discussion

The findings of this review contribute to the existing literature by addressing a research gap. The limited number of studies examining the relationship between ERT and TMDs emphasizes the importance of this review as it consolidates and critically evaluates the available evidence. This comprehensive analysis helps to overcome the limitations of individual studies and provides a more robust understanding of the association between ERT and TMDs [29].

The study of Hatch et al. [26] analysed their samples based on SES and literacy rate. Medicines consumed were also looked into by the researcher. Calibration of the examiner was done for CMI. Additionally, they also reported that women on ERT perceived self-control over their health and had greater dental consultations than their control cohorts. This observation could be because of the greater dental health insurance coverage in ERT group. In the study of LeResche et al. [27], four controls were considered intitially for every case recruited. Though this was a strong methodological point, the fact that the study included cases those who were referred for TMD pain is not to be overlooked. Also, there could be women who experienced TMD pain, but did not report to the clinics. The study of Nekora et al. [28] included women who attained menopause both naturally and surgically. Associated muscles of TMJ were also palpated. Examiner remained blind to the sample allocation of groups. All the studies had certain limitations such as not considering the duration of ERT and their current blood levels. Also, none of the studies addressed the severity of TMD.

The implications of this review are twofold. First, the results suggest that ERT does not appear to increase the risk of TMD occurrence or worsen TMD-related pain and symptoms in women. This finding is valuable for clinicians who prescribe or consider ERT for various indications, including menopausal symptoms. It provides reassurance regarding the potential effects of ERT on TMDs, which can inform treatment decisions and improve patient management. Additionally, the review highlights the need for further research in this area. The limited number of studies identified during the review process underscores the scarcity of available evidence on the specific relationship between ERT and TMDs. Future studies should aim to fill this gap by conducting well-designed prospective studies with larger sample sizes and longer follow-up periods. Additionally, it is crucial to explore the potential influence of different types and regimens of ERT on TMDs to provide a better understanding of this association [30–37]. The impact of this review on the literature is significant, as it consolidates the existing evidence and provides a comprehensive analysis of the relationship between ERT and TMDs. By highlighting the current gaps in knowledge and calling for further research, this review encourages scientific exploration in this field. Future studies inspired by this systematic review may contribute to a more substantial body of evidence, leading to a deeper understanding of the impact of ERT on TMDs and guiding clinical decision-making.

Numerous articles have explored the relationship between estrogen TMDs, yielding varying and contradictory results. In some studies, it was observed that higher estrogen levels were associated with a reduction in TMD pain [38–42]. Conversely, a couple of studies suggest that TMD was linked to elevated estrogen levels [43, 44]. However, due to insufficient data in one of the papers that we selected for our review, it remains inconclusive which subgroups of painful TMD are influenced by estrogen [28] It is important to consider that the prevalence of TMD in the studied population might have been affected by the administration of HRT, particularly ERT, primarily to women who have undergone hysterectomy. Additionally, it is worth noting that the surgical procedure itself, including endotracheal intubation, could transiently contribute to TMD pain [45]. Hence, exclusion of post-surgery patients from the study would have been beneficial to isolate the effects of estrogen on the TMJ.

Elevated estrogen levels were linked to an increased prevalence of TMD in some animal model studies [46, 47]. Estrogen's involvement in the development of TMDs can be attributed to its impact on the composition of the extracellular matrix in TMJ fibrocartilage [47]. Moreover, investigations focusing on the influence of estrogen deficiency on the TMJ have revealed that the absence of estrogen can induce pathological alterations within the joint. For instance, estrogen deficiency has been shown to diminish the synthesis of sulfated proteoglycans in articular cartilage, as evidenced in animal models [48, 49]. Additionally, estrogen can affect the synovial membrane by modulating collagen and protein content within the TMJ disc [50–52]. Collagen and elastin, major constituents of the TMJ disc, often exhibit structural changes in the presence of TMD symptoms, with sex hormones playing a significant role in regulating collagen and elastin synthesis. Following ovariectomy, an increase in the soft tissue layer and a decrease in bone volume and density within the TMJ have been observed, but restoration of these altered histomorphometric parameters can be achieved through ERT [53].

Despite its valuable contributions, this paper has several limitations that should be considered. These limitations may impact the interpretation and generalizability of the review's findings. The first is the potential for publication bias. The review process typically includes searching for published studies, and there is a possibility that studies with null or non-significant findings may not have been published, leading to an overrepresentation of studies reporting positive or significant results. This bias could affect the overall conclusions of the review and introduce an element of uncertainty. Second, the included studies in this review may have heterogeneity in terms of study design, population characteristics, assessment techniques, and reporting methods. Such heterogeneity can make it challenging to perform a quantitative synthesis and increase the risk of confounding factors influencing the results. Furthermore, the review's findings may be influenced by the specific databases searched, potentially excluding relevant studies not indexed in those databases. It is also important to note that this review focuses specifically on the association, if any existing between ERT and TMDs, and the results may not be applicable to other hormonal therapies or conditions. The generalizability of the findings to diverse populations or specific subgroups may be limited, particularly if the included studies predominantly represent a specific demographic or geographic region.

The limited number of studies highlights a notable gap in the literature evidence available assessing the relationship between TMDs and ERT. Though the current systematic review presents initial insights, future research endeavours are needed to fulfil rigorous qualifying standards in order to enhance the scientific comprehension of this intricate relationship. Well-designed investigations are imperative to enrich the knowledge base, enhance statistical power, and facilitate more accurate conclusions.

## Conclusion

This review suggests that there is a lack of consensus regarding the relationship between ERT and TMDs. While one study reported a potential increased risk of TMDs with ERT use, others did not find a significant association. The heterogeneity of the included studies and the limitations of the available evidence should be considered when interpreting these findings. The review identified limitations such as potential publication bias, heterogeneity in study designs and characteristics, varying methodological quality, and limitations in data availability. These factors highlight the need for further well-designed studies with larger sample sizes, standardized assessment techniques, and appropriate control for confounding variables to enhance the understanding of the association between ERT and TMDs. A future research perspective could focus on unravelling the molecular and cellular mechanisms through which estrogen influences the various components of the temporomandibular joint and associated structures. Future studies can also be undertaken to determine whether ERT can impact different TMD subtypes. This could facilitate more targeted therapeutic approaches based on the specific characteristics of the disorder.

## Data Availability

The data will be available on reasonable request from the corresponding author.
